# An Indoor Visible Light Positioning System Using Tilted LEDs with High Accuracy

**DOI:** 10.3390/s21030920

**Published:** 2021-01-29

**Authors:** Neha Chaudhary, Othman Isam Younus, Luis Nero Alves, Zabih Ghassemlooy, Stanislav Zvanovec, Hoa Le-Minh

**Affiliations:** 1 Instituto de Telecomunicações and Departamento de Electrónica, Telecomunicações e Informática, Universidade de Aveiro, 3810-193 Aveiro, Portugal; nero@ua.pt; 2 Optical Communications Research Group, Faculty of Engineering and Environment, Northumbria University, Newcastle upon Tyne NE1 8ST, UK; othman.younus@northumbria.ac.uk (O.I.Y.); z.ghassemlooy@northumbria.ac.uk (Z.G.); hoa.le-minh@northumbria.ac.uk (H.L.-M.); 3 Department of Electromagnetic Field, Faculty of Electrical Engineering, Czech Technical University in Prague, 16627 Prague, Czech Republic; xzvanove@fel.cvut.cz

**Keywords:** localization, visible light communication, visible light positioning, received signal strength, linear least square, polynomial regression, Tx’s tilting

## Abstract

The accuracy of the received signal strength-based visible light positioning (VLP) system in indoor applications is constrained by the tilt angles of transmitters (Txs) and receivers as well as multipath reflections. In this paper, for the first time, we show that tilting the Tx can be beneficial in VLP systems considering both line of sight (LoS) and non-line of sight transmission paths. With the Txs oriented towards the center of the receiving plane (i.e., the pointing center F), the received power level is maximized due to the LoS components on F. We also show that the proposed scheme offers a significant accuracy improvement of up to ~66% compared with a typical non-tilted Tx VLP at a dedicated location within a room using a low complex linear least square algorithm with polynomial regression. The effect of tilting the Tx on the lighting uniformity is also investigated and results proved that the uniformity achieved complies with the European Standard EN 12464-1. Furthermore, we show that the accuracy of VLP can be further enhanced with a minimum positioning error of 8 mm by changing the height of F.

## 1. Introduction

Coronavirus disease 2019 (COVID-19) has had a major impact on society at a global level, where social distancing, monitoring, and tracking has become effective in controlling and reducing the spread of the virus [1]. Precise localization and tracking technologies for use in indoor and outdoor environments will play a crucial role in dealing with COVID-19 and other pandemic outbreaks in the future. Nowadays, indoor positioning has a prominent contribution in day-to-day activities in organizations such as health care centers, airports, shopping malls, manufacturing, underground locations, etc., for the safe operating environments. In indoor environments, both radio frequency (RF) and optical wireless-based technologies could be adopted for localization [2,3]. Although the RF-based global positioning system offers higher penetration rates with reduced accuracy (i.e., in the range of a few meters), it does not work well in indoor environments (and not at all in certain cases such as tunnels, mines, etc.) due to the very weak signal and no direct access to the satellites [4,5,6]. On the other hand, the light-based system known as a visible light positioning (VLP) system, which uses the light-emitting diodes (LEDs)-based lighting infrastructure, could be used at low cost and high accuracy compared with the RF-based system [7,8].

VLP can be implemented using different techniques. Proximity and scene analysis (i.e., fingerprinting) are considered the simplest methods with relatively low positioning errors *ε_p_* i.e., typically in a range of 10 to 45 cm, depending on the fingerprint database [8,9,10]. In the scene analysis technique, the estimation process of the relative position can be obtained by comparing the measured value with a pre-measured location of each position and then matching it to determine the real position. However, the measurement can be affected by the distributions of base stations, i.e., transmitters (Txs), shadowing and blocking, as well as the absolute location (i.e., probabilistic and correlation) dependency on pattern recognition techniques [9]. A VLP using two photodiodes (PDs) and an image sensor (IS) was proposed in [7,8,11]. Note, visible light communication (VLC) with IS (composed of a large PD array) naturally fits well with multiple inputs multiple-output systems in indoor and outdoor applications. In IS-based VLP, image-processing techniques can be used to determine the position but at the cost of increased complexity [12]. Note that, in VLP the transmission speeds (i.e., data rates) of the PD and IS are not critical at all since the aim is to achieve positioning with high accuracy [13]. Most research reported on VLP has focused on the investigation of geometrical properties using triangulation/trilateration, fingerprinting, or proximity methods to determine the transmission distance based on establishing a one-to-one relationship between the target location and its received signal strength (RSS). In such works, the analyses were based on the intensity modulation, angle of arrival [9], time of arrival [10], time difference of arrival [14], time of flight (TOF), and direct detection. In VLP systems, linear least square (LLS) or non-linear least square (NLLS) algorithms are often used for the position estimation [15,16,17].

Despite the fact that the user’s mobility can influence the performance of the VLP system, most research reported in the literature has focused primarily on static scenarios. The major issues of shadowing and blocking affecting user’s mobility were reported in [18], where the VLC system performance considering the changes in the channel conditions in different indoor scenarios (i.e., a furniture equipped office room, an empty hall, and a corridor) was investigated. It was shown that, the cumulative distribution function (CDF) of the received power distribution differs in the worst case by up to 7% in a furnished office (people density > 0.16 people/m^2^). Alternatively, the highest root mean square (RMS) delay spread of 6.5% in comparison with the case with no people was observed for an empty hall. The results also revealed that, the corridor with the maximum RMS delay of 2% at the people density > 0.16 people/m^2^ is the most robust against the people’s movement compared with the other two where the problem of shadowing or blockage could be readily avoided. Another concern with the user’s mobility is the processing time required that needs considering with respect to the speed of movement for the receiver (Rx).

In most of the reported methods, the angular dependency was neglected in RSS-based localization with the assumption that, the Rx has a fixed height and is pointing up towards the Txs [19]. However, computational and implementation costs are too high, and the assumptions made may not be valid in real-time application scenarios with mobile Rxs, which needs further investigation. Recent works have focused on the impact of multipath induced reflections on the performance of VLP without considering the tilting angles [20,21,22], where it was shown that, multipath reflections considerably increase *ε_p_*; whereas in [23], it was shown that, the channel capacity can be significantly improved by carefully selecting the Rx’s tilting angle $\theta_{\mathrm{Rx}}$. However, the initial research demonstrated that in VLP $\theta_{\mathrm{Rx}}$ usually results in increased *ε_p_* (i.e., lower accuracy).

The widely used commercially available LED spotlights in building facilitates the concept of using Txs with tilting features. For instance, the impact of the Tx (LED) tilting angle $\theta_{\mathrm{Tx}}$ on the accuracy of RSS-based VLP was studied in [24], where it was shown that *ε_p_* increased (i.e., in the order of centimeters) with $\theta_{\mathrm{Tx}}$. In [25], a 4-LED VLP system using an artificial neural network (ANN) was proposed to improve the positioning accuracy, which is impacted by the random and unknown static Tx tilt angle with a maximum variation of 2°. It was shown that ANN offered improved performance compared with standard trilateration, achieving localization errors below 1 cm for the line-of-sight (LoS) channel. In Addition, an RSS-based localization algorithm with multidimensional LED array was proposed in [26], where the design of the lamp structure was introduced to exploit the direction of the LED in a LoS environment. The authors showed that, the proposed system achieved a RMS error of 0.04 and 0.06 m in two- and three-dimensional localization, respectively for the LED with a tilt angle of 15°. While in [27], an angle diversity Tx (ADT) together with accelerometers was proposed for uplink three-dimensional localization in a LoS environment. ADT was a combination of 19 or 37 LEDs (LEDs array), which were placed on the ground, and PDs located on the ceiling. The results showed that, an average localization error of less than 0.15 m. 

The impact of non-line of sight (NLoS) path in a VLC system deployed in a referenced empty room has been reported in the literature. In [28], the impact of the power levels from NLoS paths on the performance of VLP for different Rx positions and their orientations was reported. It provided a theoretical framework for the design of VLP resource allocation methods to improve the performance of the non-tilted Tx. Channel modeling and its characterisation with the existence of reflections from objects and surfaces were investigated in [29]. Considering the delay spread and the channel gain in a typical room, it was shown that it is not required to consider all objects within rooms [29,30]. Moreover, the use of flexible organic LED-based VLC in indoor environments (i.e., offices, corridors, semi-open corridors in shopping malls, etc.) was investigated in [31], where it was shown that the channel gain in an empty room is higher by 4.8 and 5.2 dB compared with the fully furnished room and a semi-open corridor, respectively [31].

Unlike previous works, in this paper we investigate LED tilting for the first time and show that it can be beneficial in VLPs in improving the positioning accuracy (PA). We show the impact of reflections on the accuracy by means of the received power from both LoS and NLoS transmission paths, the positioning algorithm utilized, and the accuracy of the VLP system for a single PD-based static Rx (i.e., putting the Rx at fixed locations) where the user movement has not been considered. In this approach, the Txs are oriented towards the pointing center F with the (*x_F_*, *y_F_*, *z*_F_) coordinates without violating the acceptable uniformity range of the light distribution in the illuminated region. Note, F is selected at the center of the receiving plane in this work, and alignment is achieved with respect to the Tx normal ${\hat{t}}_{k}$. 

We investigate the regression, which is fitted with the received power *P_R_* points at various Rx locations for two different scenarios. Note, the Rx locations are within a squared shape region centered at F with a side length *D_r_*. The polynomial regressions (PRs) are fitted with the PR points for the full and half rooms of areas of 6 × 6 and 3 × 3 m^2^, which is termed as scenarios S1 and S2, respectively-. The study is carried out using the LLS algorithm for position estimation, which is a low complexity solution. Hence, we offer a significant accuracy improvement by up to ~66% compared with a link without Tx’s tilt. We show *ε_p_* of 1.7, and 1.3 cm for S1 and S2, respectively, and for *z*_F_ of 0 m (i.e., the height of F from the floor level). Furthermore, we investigate *z*_F_ with respect to *ε_p_* and we show that, the lowest *ε_p_* of 1.3 and 0.8 cm were for S1 and S2, respectively.

The remainder of this paper is structured as follows. Section 2 presents the VLC system model used in the positioning algorithm. The positioning algorithm is briefly explained in Section 3. The results and discussion are included in Section 4. Finally, Section 5 provides the conclusion of the paper.

## 2. Proposed Visible Light Positioning (VLP) System Model

**Figure 1:**
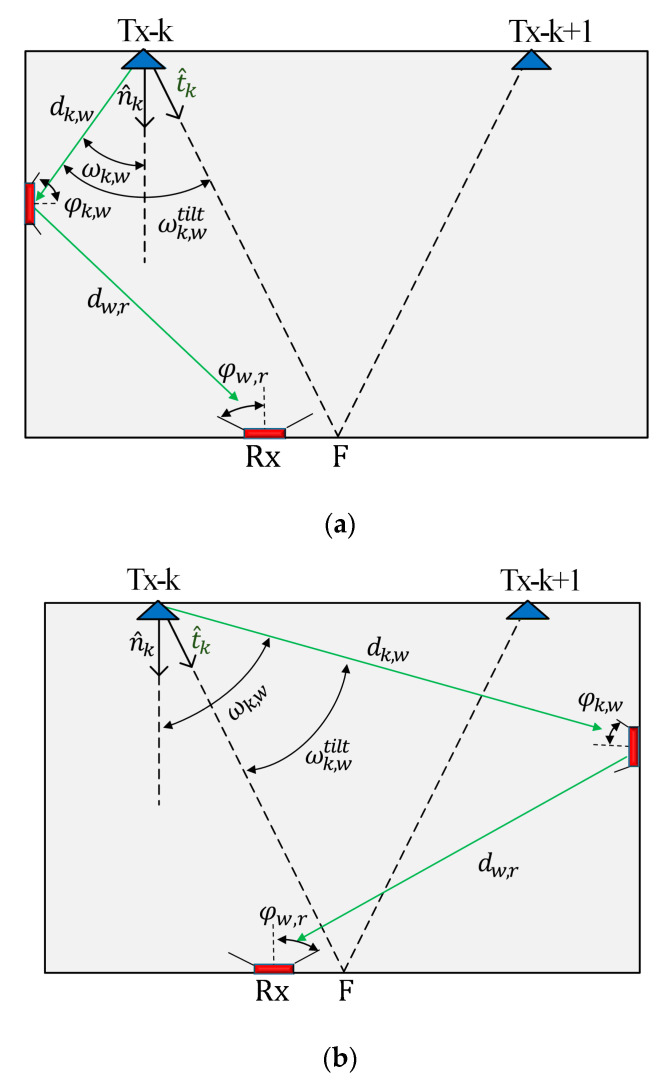
An example of a reflected light ray in case of light-emitting diode (LED) tilt: (**a**) near-wall reflections case, and (**b**) far wall reflections case.

In RSS-based localization systems, positioning accuracy depends mainly on *P_R_*. For NLoS links, reflection from near and far walls should be considered, which contributes to the degradation of PA. For example, Figure 1 illustrates a system with two Txs aligned with respect to F (i.e., shown as the tilted Tx normal ${\hat{t}}_{k}$), which is used to investigate the impact of reflections from walls on the accuracy of VLP). Here, the aim is to maximize *P_R_* from the LoS paths to improve accuracy at F, which is initially set at the center of the receiving plane (i.e., $x_{F},\;y_{F},$ and $z_{F}$ are all set to zero). The tilting orientation is estimated based on the position of F, which is given by:

(1) $${\hat{t}}_{k}=\frac{\vec{T_{k}}}{\left\|\vec{T_{k}}\right\|},$$

where $\vec{T_{k}}$ is a vector that represents the difference between the coordinates of the *k*th Tx and point F $(x_{F},y_{F},z_{F}),$ and ‖·‖ is the Euclidean norm. The tilted irradiance angle $\omega_{k,w}^{\mathrm{tilt}}$ is given by:

(2) $$\cos\left(\omega_{k,w}^{\mathrm{tilt}}\right)=\frac{d_{k,w}\cdot {\hat{t}}_{k}}{\left\|d_{k,w}\|\cdot \|{\hat{t}}_{k}\right\|},$$

where $d_{k,w}$ is the distance between the *k*th Tx and the reflective area, and · represents the product dot operation.

The NLoS power contributions from the near-wall reflections represented by the Tx’s cosine terms expressed in (2) can be reduced by tilting the Txs towards F (i.e., ${\hat{t}}_{k}$ is directed towards F that implies $\omega_{k,w}^{\mathrm{tilt}}>\omega_{k,w}$, where $\omega_{k,w}$ is the irradiance angle with no tilted Tx, see Figure 1a. Even though the Tx’s cosine terms of NLoS signals will increase for the far-wall reflections, which is implied by $\omega_{k,w}^{\mathrm{tilt}}<\omega_{k,w}$, the link experience a higher path loss due to the longer transmission range, see Figure 1b. Having these observations in mind, we can infer that tilting the Txs can be beneficial in VLP by leveraging the effect of reflections from both near- and far-walls. Under this perspective, it reasonable to explore tilting based on F at the center of the receiving plane and investigate how this can improve PA. These observations remain valid for the entire area of the walls when concerning the first reflection. Higher-order reflections also have an impact on positioning accuracy. However, due to the fact that these higher-order reflections have reduced power levels when compared with the LoS and 1st order case in regions near the center of the room, the previous discussion is still valid, and LoS power can be maximized by tilting towards the center.

**Figure 2:**
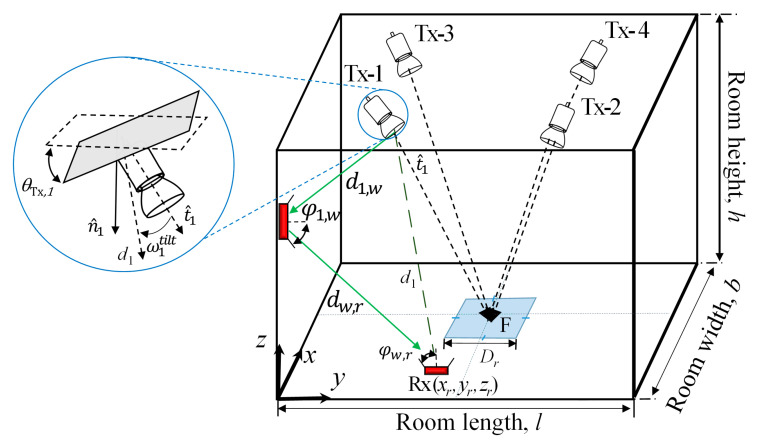
The proposed indoor visible light positioning (VLP) system with the tilted transmitter (Tx).

Figure 2 shows the geometrical set-up diagram of the proposed indoor VLP system, which is composed of 4 Txs (i.e., LEDs) and an Rx (i.e., a PD) positioned on the ceiling and the floor level, respectively. Each *k*th Tx has a known set of coordinates (*x_k_*, *y_k_*, *z_k_*), which is associated with the world coordinate system (WCS), with ${\hat{t}}_{k}$ of $\left[{\sin\theta }_{\mathrm{Tx},k}\;\cos\alpha_{k},{\;\sin\theta }_{\mathrm{Tx},k}\;\sin\alpha_{k},\;-{\cos\theta }_{\mathrm{Tx},k}\right]$ where $\theta_{\mathrm{Tx},k},\;\alpha_{k}$ are the tilting and azimuth angles, respectively and *k* is 1, …, 4. Note that, in this work, as a reference, an empty room is considered to study the impact of Tx’s tilting on the positioning accuracy. The proposed system can be utilized for positioning purposes where the positioning accuracy is a major concern. However, if indoor positioning system uses the already existing wireless communication network architectures, then high accuracy may no longer be critical. Therefore, there exists always a trade-off between accuracy and other system requirements including scalability, complexity, coverage, etc.

**Figure 3:**
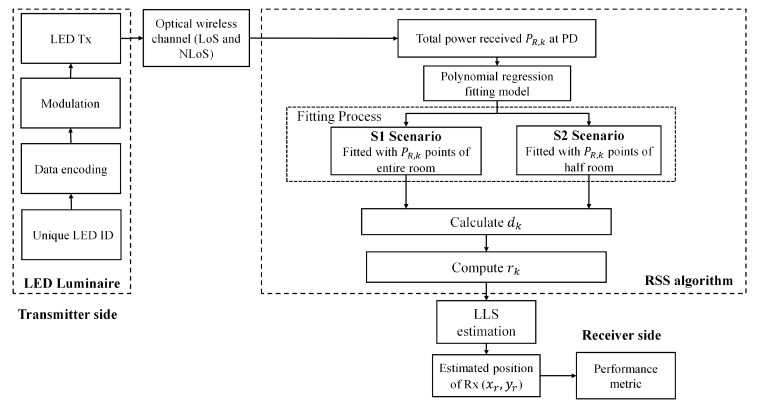
Block diagram of the proposed VLP system.

Each Tx broadcast unique ID information of 2 bits, which is encoded and modulated using on-off keying (OOK), which allows separation at the Rx using a correlation method that can be received at the Rx in advance of location identification, see Figure 3. Considering the 1st order reflections, the received total power is given by:

(3) $$P_{R}=\sum P_{R-\mathrm{LoS}}+\sum P_{R-\mathrm{NLoS}},$$

where $P_{R-\mathrm{LoS}}$ and $P_{R-\mathrm{NLoS}}$ represent the received power for LoS and NLoS, respectively. Typically, the signal-to-noise ratio in standard VLC will be high (>20 dB [32]), which would be considered noise-free in common cases). Moreover, noise sources (mostly dominated by the background lights) [32] will have a similar effect on the VLP system with and without tilting Tx. Thus, a noise-free system is considered in this work. The conventional trilateration technique based on a range of three minimum observation points offers the advantage of simple geometrical solutions [14]. Using the RSS algorithm and 4-Tx (i.e., LEDs), the $P_{R-\mathrm{LoS}}$ for the LoS path is given as [33,34]:

(4) $$\sum P_{R-\mathrm{LoS}}=\overset{K}{\underset{k=1}{\sum }}C_{o}P_{t}\frac{\cos^{m}\left(\omega_{k}^{\mathrm{tilt}}\right)\;\cos\left(\varphi \right)}{{\left\|d_{k}\right\|}^{2}}T_{s}\left(\varphi \right)g\left(\varphi \right),$$

where

(5) $$C_{o}=\frac{m+1}{2\pi }ℛ\;A_{r},$$

and

(6) $$m=-\frac{\ln\left(2\right)}{\ln\left(\cos\left(\Theta_{\frac{1}{2}}\right)\right)},$$

where *K* is the total number of Txs, $\Theta_{1/2}$ is the light source irradiance half-power angle, $\omega_{k}^{\mathrm{tilt}}$ and *φ* are the tilted irradiance angle from the *k*th Tx to the Rx and the receiving incident angle, respectively. $d_{k}$ is the distance between *k*th Tx and Rx. *A_r_* and ℛ are the PD’s active area and responsivity, respectively. $T_{s}\left(\varphi \right)$ and g(φ) are the gains of the optical filter and the concentrator at the Rx, respectively. Note, $T_{s}\;\left(\varphi \right)$ and g(φ) are set to unity, *φ* < 90° and *d* ≫ $\sqrt{A_{r}}$.

For the NLoS path and considering only the first-order reflection, the received total power can be expressed as [32]:

(7) $$\sum P_{R-\mathrm{NLoS}}=\overset{K}{\underset{k=1}{\sum }}\underset{\mathrm{wall}}{\sum }\rho C_{o}P_{t}A_{\mathrm{ref}}\frac{\cos^{m}\left(\omega_{k,w}^{\mathrm{tilt}}\right)\;\cos\left(\varphi_{k,w}\right)}{\pi {\left(\left\|d_{k,w}\|\|d_{w,r}\right\|\right)}^{2}}\;T_{s}\left(\varphi_{w,r}\right)\;g\left(\varphi_{w,r}\right)\cos\left(\omega_{w,r}\right)\cos\left(\varphi_{w,r}\right),$$

where $d_{k,w}$, $\omega_{k,w}^{\mathrm{tilt}}$, and $\varphi_{k,w}$ are the distances, irradiance angle, and the receiving incident angle between the *k*th Tx and the reflective area, respectively. $d_{w,r}$, $\omega_{w,r}$, and $\varphi_{w,r}$ are the distances, irradiance angle, and the receiving incident angle between the reflective area and the Rx, respectively, see Figure 1a. *ρ* is the reflection coefficient, which depends on the material of the reflective surface and $A_{\mathrm{ref}}$ is the reflection area. $P_{R-\mathrm{NLoS}}$ for the signals from the NLoS paths is determined based on the Matlab code 3.2 from [32].

Moreover, the uniform distribution of the $P_{R}$ inside the illuminated zone is essential in indoor environments [16]. The uniformity of light distribution in the room (*U*) is represented as the ratio of the minimum to maximum power intensity at the receiving plane, which is given by:

(8) $$U=\frac{\min\left(P_{R}\right)}{\max\left(P_{R}\right)},$$

Here we consider a grid (1 cm resolution) of 3600 Rx positions on the receiving plane, which is associated with WCS of (*x_r_*, *y_r_*, *z_r_*). We have also specified the dedicated region, which is a square shape centered at the point F and located at the receiving plane. The receiving positions are considered inside this region only. All the other key system parameters are given in Table 1.

## 3. Positioning Algorithm

### 3.1. Distance Estimation Using Polynomial Regression

The block diagram of the proposed VLP system is shown in Figure 3, in which $P_{R}$ is processed to estimate the Rx position. Distance estimation is the central feature of the RSS positioning approach, and for LoS paths it is normally deducted from (4), which is estimated as:

(9) $${\left\|d_{k}\right\|}^{2}={\left\|r_{k}\right\|}^{2}+h^{2}$$

where *h* is the vertical distance between the Tx and the Rx. The estimated distance between the Rx and the *k*th Tx can be estimated from (4), which is given by:

(10) $$r_{k}=\sqrt{{\left(\frac{P_{t}C_{o}h^{m+1}}{P_{R-\mathrm{LoS},k}}\right)}^{\frac{2}{m+3}}-h^{2},}$$

where $P_{R-\mathrm{LoS},\;k}$ is the LoS received power at Rx from *k*th Tx. In NLoS links, this approach results in increased errors due to reflections [35,36], therefore the distance estimation approach using (10) is no longer valid. One possible approach would be to generate a polynomial fitted model for the power and distance relationship as defined by:

(11) $$d_{k}=a_{0}+a_{1}P_{R,k}+a_{2}{\left(P_{R,k}\right)}^{2}+\cdots +a_{j}{\left(P_{R,k}\right)}^{j},$$

where *a_j_* is the coefficient of the fitted polynomial at *j*th degree polynomial and $P_{R,\;k}$ is the total received power at Rx from *k*th Tx. Note, $d_{k}$ is computed using (11), which is then substituted into (9) to determine $r_{k}$.

### 3.2. Linear Least Square (LLS) Estimation

LLS is adopted to analyze the performance of the proposed system by considering the estimated distances of the NLoS paths, which is a low complexity solution as compared with the NLLS algorithm. Following geometric properties, a minimum of 3-Tx located at the center of the circle is required, where the estimated distance is considered as the circle radius. The intersection point of the three circles is considered as the measured position of the Rx. E.g., the *k*th LED luminaire is positioned at (*x_k_*, *y_k_*, *z_k_*) and the Rx is located at (*x_r_*, *y_r_*, *z_r_*). A closed-form solution using the LLS estimation method is given by:

(12) $$X={\left(A^{T}A\right)}^{-1}A^{T}B$$

where

(13) $$A=\left[\begin{array}{cc} x_{2}-x_{1} & y_{2}-y_{1}\\ x_{3}-x_{1} & y_{3}-y_{1}\\ x_{4}-x_{1} & y_{4}-y_{1} \end{array}\right],\;\;\;X=\left[\begin{array}{c} x_{r}\\ y_{r} \end{array}\right]$$

(14) $$B=0.5\times \left[\begin{array}{c} \left(r_{1}{}^{2}-r_{2}{}^{2}\right)+\left(x_{2}{}^{2}+y_{2}{}^{2}\right)-\left(x_{1}{}^{2}+y_{1}{}^{2}\right)\\ \left(r_{1}{}^{2}-r_{3}{}^{2}\right)+\left(x_{3}{}^{2}+y_{3}{}^{2}\right)-\left(x_{1}{}^{2}+y_{1}{}^{2}\right)\\ \left(r_{1}{}^{2}-r_{4}{}^{2}\right)+\left(x_{4}{}^{2}+y_{4}{}^{2}\right)-\left(x_{1}{}^{2}+y_{1}{}^{2}\right) \end{array}\right].$$

## 4. Results and Discussion

### 4.1. Impact of the Transmitter (Tx) Tilting on the Radiation Pattern

**Figure 4:**
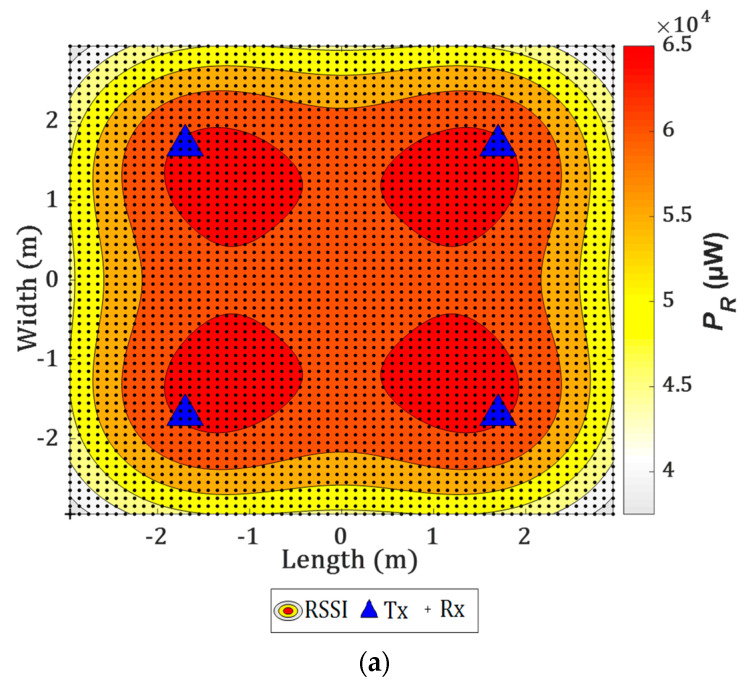

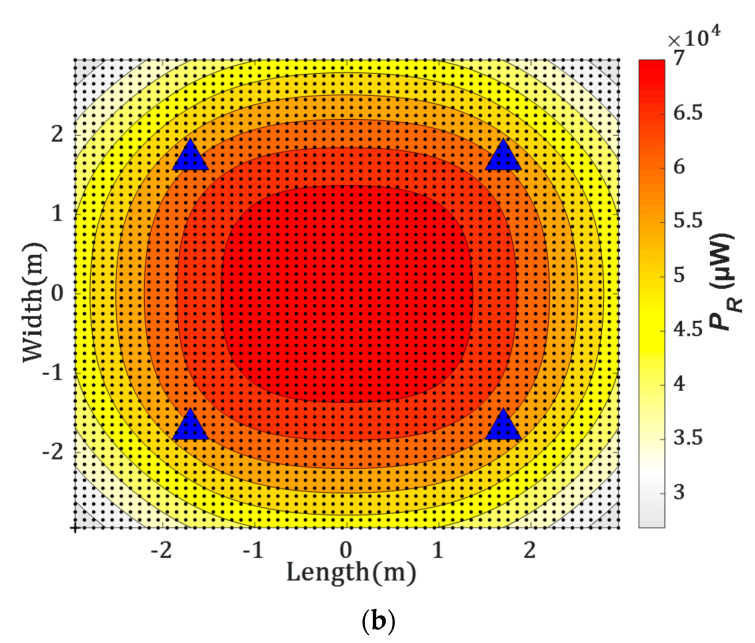
The received power distributions for the proposed system for the Txs with: (**a**) no tilting, and (**b**) tilting.

Figure 4a shows the received power distributions for the link (i.e., received signal strength indicator RSSI) with and without the tilting Txs. Note, the Txs are directed towards F following the proposed model in Section 2. As shown in Figure 4b, there is a significant improvement in the power distribution with the tilting Txs (i.e., a much more uniform distribution) around the center of the receiving plane. All the observed tilted Tx normal ${\hat{t}}_{k}$ for 4-Tx are given in Table 2.

### 4.2. Polynomial Fitting

With reference to Figure 3, $d_{k}$ is estimated based on *P_R,k_* and the PR (polynomial regression) method as outlined in Section 3.1. The accuracy and precision of fitting are measured by the coefficient of determination R^2^, which is a statistical measure of how close the data are to the fitted regression line, and the standard deviation. Note, PR is considered for various data points and categorized into two scenarios S1 and S2 based on the room dimensions. For scenarios S1 and S2, the PRs are fitted with the *P_R,k_* points for the full and half rooms of areas of 6 × 6 and 3 × 3 m^2^, respectively. The deviation of *P_R,k_* points is impacted mainly by the reflections wherein the data near the walls imply a larger estimation error as stated previously in the literature [19,32]. Therefore, 3600 samples (a full room with a 1 cm grid size) are considered for the polynomial fitting for S1, while for S2 we only have considered 900 samples (an inner half room). A stabilized residual sum of squares is achieved with the polynomial order *j* of 4. The polynomial coefficients of the fitted curve and R^2^ are estimated for both S1 and S2.

**Figure 5:**
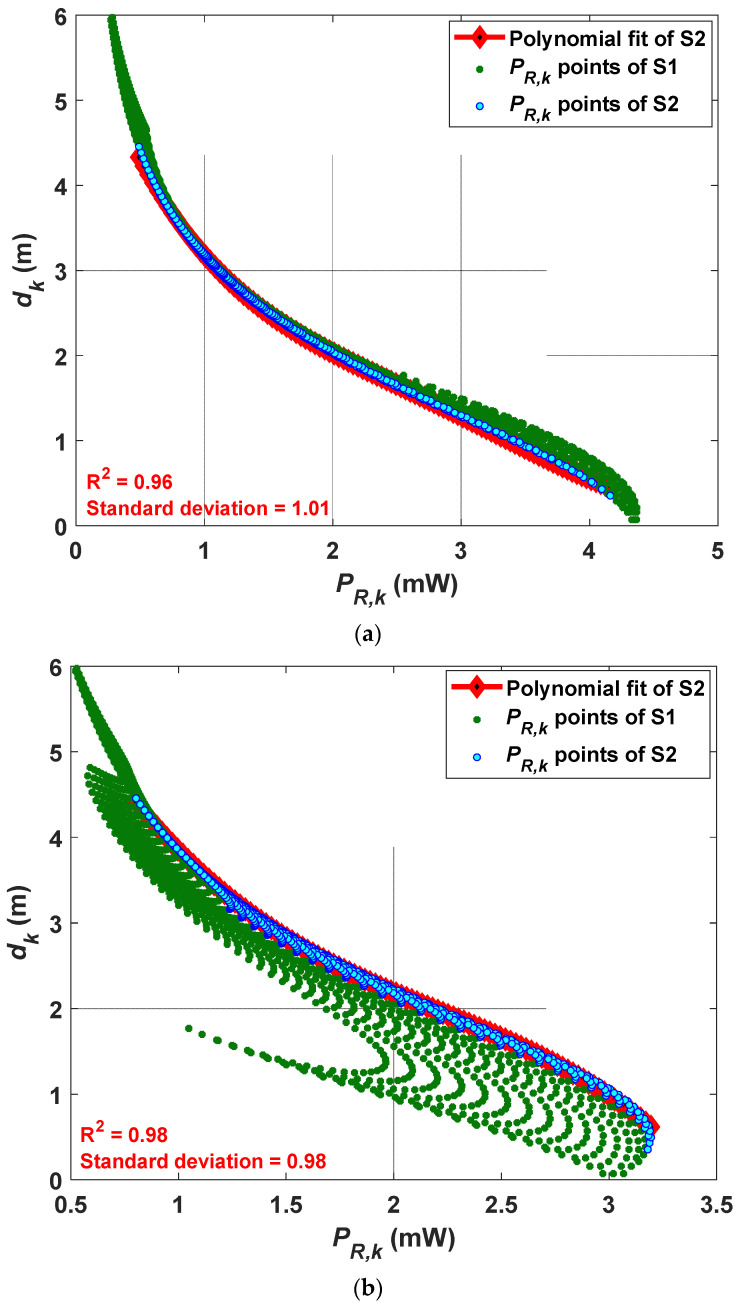
The distance estimation for Tx-k using the polynomial regression (PR) method employed in S2 for the Txs with: (**a**) no tilting, and (**b**) tilting.

The polynomial fitted curves for VLP without and with the tilting Txs are illustrated in Figure 5. The green points and blue plots indicate the *P_R,k_* points for the full and half rooms, respectively. Figure 5a shows that, the *P_R,k_* points span between 0 and 4.2 mW, and are uniformly distributed for both S1 and S2. However, Figure 5b depicts that the *P_R,k_* points for S1 are more scattered with a smaller span of 0.5 to 3.2 mW, which corresponds to the corner of the room. In S2, the *P_R,k_* points are more focused towards S2 due to tilting of the Tx, thus the fitting data points are considered for S2 only. From the results obtained, both R^2^ and the standard deviation are positively affected with tilting of the Tx, i.e., higher R^2^ value of 0.98 and lower standard deviation of 0.98 is achieved for the tilted Tx as compared with a lower R^2^ value of 0.96 and higher standard deviation of 1.01 in the case of no tilted Tx, see Figure 5b. Table 3 shows the estimated polynomial coefficients and R^2^ values for S2 with and without the tilted Txs.

### 4.3. Impact of the Tx Tilting and the Altitude of F on VLP

**Figure 6:**
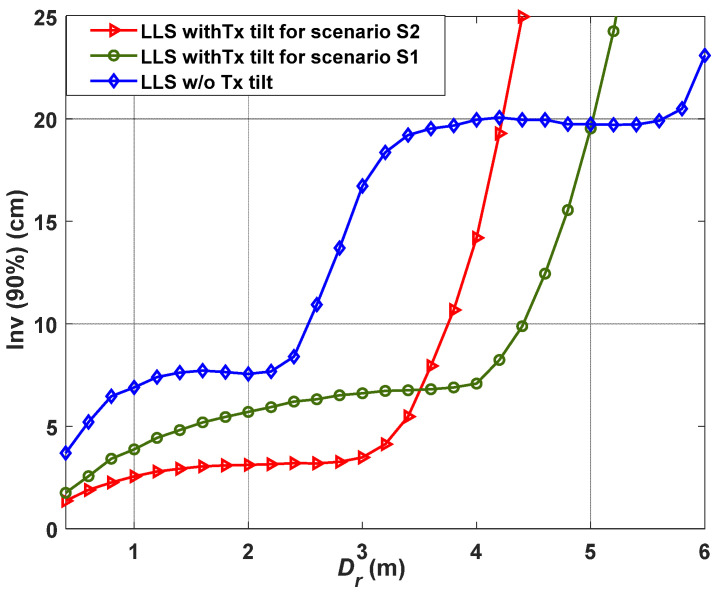
The measured quantile function at χ of 90% for various *D_r_* for linear least square (LLS) with and without the tilted Txs.

In this section, we investigate *ε_p_* for different values of *D_r_* to realize the impact of tilted Txs near the center of the receiving plane, and further analyze the impact of changing the height of *z*_F_ on the positioning accuracy. Figure 6 illustrates Inv(90%) as a function of *D_r_* for S1 and S2 with the LLS algorithm, which is applied for the case with LoS and NLoS paths to estimate the Rx’s position, as described in Section 3. The quantile function Inv(χ) is used as a performance metric to observe the confidence interval of *ε_p_*, which is given by:

(15) $$\varepsilon_{p,\chi }=\mathrm{Inv}\left(\chi \right)={\mathrm{CDF}}^{-1}\left(\chi \right)$$

where χ is the percentage of the confidence interval, and CDF represents the cumulative distribution function of *ε_p_*.

To ensure a VLP link with high reliability, we have selected a 90% confidence interval for *ε_p_* to include the majority of the measured points. Note that, the Txs’ tilting angle is fixed at the point F for all values of *D_r_*. Moreover, the error can be reduced significantly depending on S1 or S2. For instance, for S1, *ε_p_* values of 1.7 and 3.6 cm are obtained for both tilting and non-tilting scenarios, respectively for *D_r_* of 40 cm. In addition, we have achieved the accuracy improvement of 44, 24, 60, and 64% for *D_r_* of 1, 2, 3, and 4 m, respectively with the maximum accuracy improvement of 66% for *D_r_* of 3.6 m. In addition, for S2, *ε_p_* of 1.3 cm is obtained for the observation area with *D_r_* of 40 cm with the tilted Tx. Hence, the Tx’s tilting (LED tilting angle) can improve the positioning accuracy in both S1 and S2 with the same detection area of 5 × 5 m^2^ (up to *D_r_* of 5 m) as compared with the case with non-tilting Tx. This could be explained by the fact that, for large observation areas (i.e., large *D_r_*), the CDF of the error becomes affected by the walls and corners of the room, with no improvement in the accuracy. Hence, the NLoS paths become dominant for regions far away from the point F, which degrades the positioning accuracy. Therefore, the proposed VLP system with the tilted Txs outperforms the system with no tilting Txs for almost the entire room i.e., an area of 5 × 5 m^2^.

**Figure 7:**
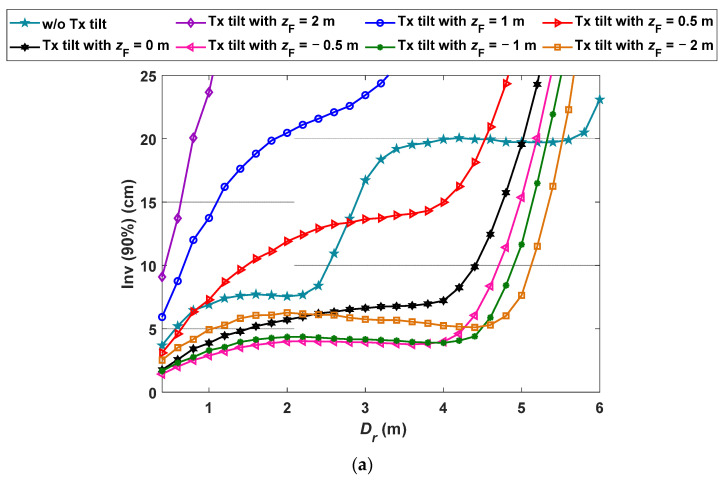

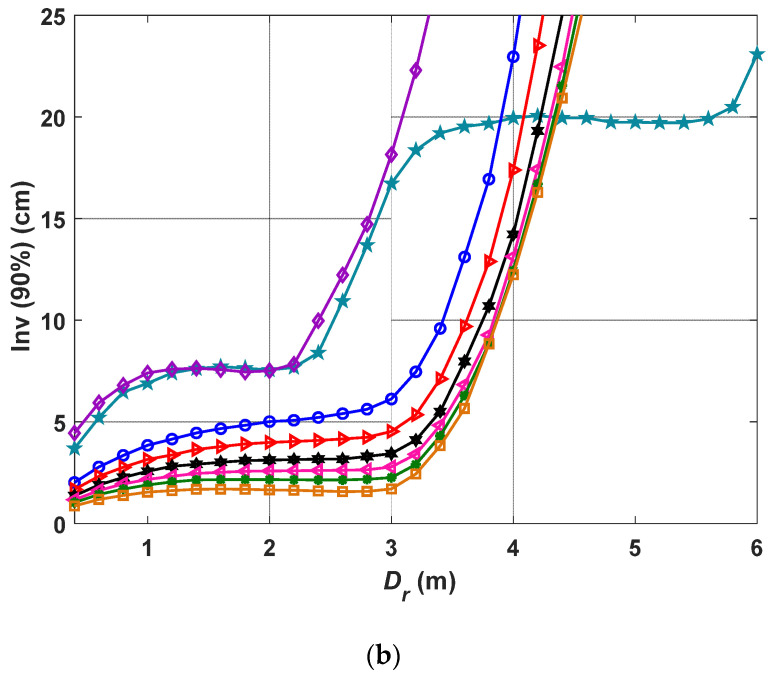
The measured quantile function at χ of 90% for various *z*_F_ values for: (**a**) S1, and (**b**) S2.

We further analyze the impact of changing the height of pointing center F (i.e., *z*_F_) on the positioning accuracy, which is eventually the variation in the Tx’s tilting. Figure 7 depict the Inv(90%) as a function of *D_r_* for a range *z*_F_ (i.e., −2 to 2 m) with and without the tilting Txs for S1 and S2. Note that, a high negative value of *z*_F_ implies that the Tx is pointing vertically downwards towards the Rx. For instance, −∞ for *z*_F_ corresponds to the standard non-tilted case and it does not imply reception under the floor. From the Figure 7, it is observed that, (i) *ε_p_* increases and decreases s with the positive and negative values of *z*_F_ (i.e., *z*_F_ > 0, < 0), respectively for both S1 and S2; (ii) the minimum *ε_p_* of 1.3 cm is at *z*_F_ of −0.5 m compared with 1.7 cm for *z*_F_ of 0 m for S1 with *D_r_* of 40 cm, see Figure 7a; and (iii) the lowest *ε_p_* is achieved at −2 < *z*_F_ < 0 m depending on the value of *D_r_*. The proposed VLP system can be further improved for the regions with *D_r_* of up to 5.5 m by adjusting the negative value of *z*_F_. For S2, the minimum *ε_p_* of 0.8 cm is observed at *z*_F_ of −2 m and *D_r_* of 40 cm compared with 1.3 cm at F (i.e., *z*_F_ = 0 m), see Figure 7b. However, the case with tilting Txs offers the lowest *ε_p_* for *D_r_* up to 4.36 m.

**Figure 8:**
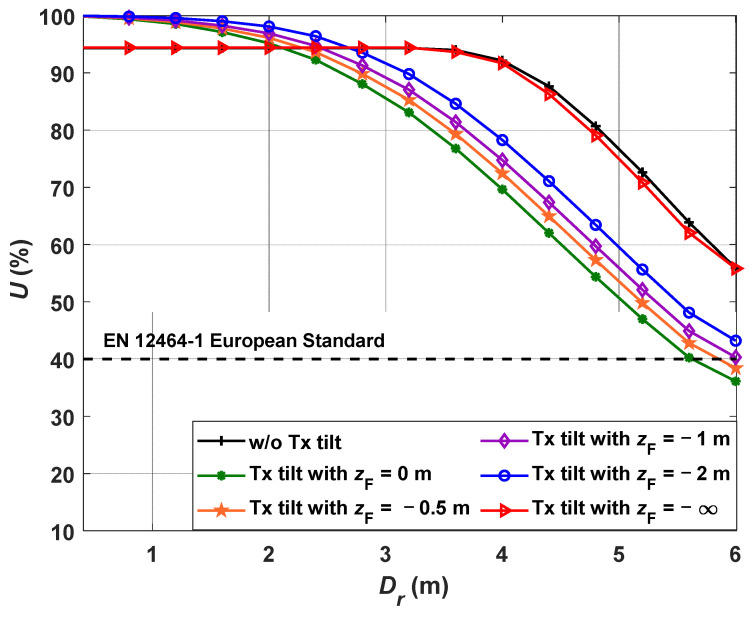
The uniformity of light distribution in different *D_r_ w*/*o* and with the tilting Txs.

Finally, Figure 8 shows the uniformity of light distribution *U* against *D_r_* without and with the tilting Tx and a range of *z*_F_. The dashed line represents the EN 12464-1 European standard of lighting in an indoor environment [37], which defines the minimum acceptable ranges of uniformity of the light distribution. We have shown that the proposed VLP system with the tilting Txs is capable of providing higher uniformity for the entire room for *z*_F_ ≤ −1 m. The uniformity of the VLP system with tilted Tx increases with the decreased value of *z*_F_.

## 5. Conclusions

In this paper, a novel approach was proposed to achieve a highly accurate indoor VLP system by considering multipath reflections. Initially, the Tx was tilted towards the center of the receiving plane to achieve higher accuracy by maximizing the received power level due to contributions from the LoS paths at the pointing center F. The positioning error was estimated by using the LLS algorithm with polynomial regression. We investigated the regression fitted with the received power points for two scenarios of S1 and S2. The results showed a significant improvement in the accuracy by up to ~66% compared with a typical non-tilting Tx case. In addition, positioning errors of 1.7, and 1.3 were obtained for the tilted Tx for S1 and S2, respectively at *z*_F_ of 0 m. The results also showed that, the uniformity of the proposed VLP system in line with European Standard EN 12464-1, therefore meeting the uniformity requirement of the visible illumination regions. Furthermore, we improved the accuracy of the proposed VLP system by controlling the height of F by achieving the lowest *ε_p_* of 1.3 and 0.8 cm for S1 and S2, respectively. Ultimately, it was concluded that the proposed VLP system with the tilting Tx outperforms the non-tilted Tx scenario. Likewise, we could gain lower *ε_p_* when considering S2, whereas *ε_p_* will increase with *D_r_* as indicated for S1.

## Figures and Tables

**Table 1:** The key system parameters.

Parameter	Symbol	Value
Room size	(*l*, *b*, *h*)	6 × 6 × 3 m^3^
The coordinates of		
Tx-1	(*x*_1_, *y*_1_, *z*_1_)	(−1.7 m, −1.7 m, 3 m)
Tx-2	(*x*_2_, *y*_2_, *z*_2_)	(1.7 m, −1.7 m, 3 m)
Tx-3	(*x*_3_, *y*_3_, *z*_3_)	(−1.7 m, 1.7 m, 3 m)
Tx-4	(*x*_4_, *y*_4_, *z*_4_)	(1.7 m, 1.7 m, 3 m)
Transmit power of each Tx	$P_{t}$	1 W
Receiver’s field of view	FoV	75°
Reflection coefficient	ρ	0.7
Half power angle	HPA	60°
Photodiode area	$A_{r}$	10^−4^ m^2^
Responsivity	ℛ	1 A/W
Reflection coefficient	ρ	0.7

**Table 2:** The values of tilted Tx normal for all Txs.

Tx Number	$\mathrm{Tilted}\;\mathrm{LED}\;\mathrm{Normal},\;{\hat{t}}_{k}$
Tx-1	[0.4, 0.4, −0.8]
Tx-2	[−0.4, 0.4, −0.8]
Tx-3	[0.4, −0.4, −0.8]
Tx-4	[−0.4, −0.4, −0.8]

**Table 3:** The coefficients of the polynomial fitted curve for the scenario S2.

Cases	Estimated Polynomial Coefficients (No Units)					R^2^
	$a_{0}$	$a_{1}$	$a_{2}$	$a_{3}$	$a_{4}$	
With tilted Tx	7.38 × 10^4^	−3.60 × 10^5^	2.37 × 10^4^	−6.26 × 10^2^	8.10	0.98
Without tilted Tx	8.86 × 10^6^	9.93 × 10^5^	3.96 × 10^4^	7.35 × 10^2^	7.44	0.96

## Nomenclature

Short Form	Description
ADT	Angle diversity transmitter
ANN	Artificial neural network
CDF	Cumulative distribution function
IS	Image sensor
LEDs	Light-emitting diodes
LLS	Linear least square
LoS	Line of sight
NLLS	Nonlinear least square
NLoS	Non-line of sight
OOK	On-off keying
PA	Positioning accuracy
PDs	Photodiodes
PR	Polynomial regression
RF	Radio frequency
RMS	Root mean square
RSS	Received signal strength
RSSI	Received signal strength indicator
Rx	Receiver
TOF	Time of flight
Tx	Transmitter
VLC	Visible light communication
VLP	Visible light positioning
WCS	World coordinate system
